# Cannabidiol decreases histological intestinal injury in a neonatal experimental model of necrotizing enterocolitis

**DOI:** 10.1186/s42826-024-00211-9

**Published:** 2024-06-27

**Authors:** Nerea Huertos Soto, Juan Manuel Gómez Cervantes, María Jesús Fernández Aceñero, María del Carmen Soto Beauregard

**Affiliations:** 1 Physiopathology and neurological therapy of INA (NEURO-INA-IN). Health Research Institute of the Hospital Clínico San Carlos (IdISSC). Community of Madrid’s Youth Employment Program (PEJ-2021 AI/BMD 21347), Madrid, Spain; 2 Pediatric Surgery Department, San Carlos Clinical Hospital, 6th south, Profesor Martín Lagos, s/n, Madrid, 28040 Spain; 3 Health Research Institute of the Hospital Clínico San Carlos (IdISSC), Madrid, Spain; 4 Pathology Department, San Carlos Clinical Hospital, Madrid, Spain; 5 Health Research Institute of the Hospital Clínico San Carlos (IdISSC), Madrid, Spain

**Keywords:** Enterocolitis, Cannabidiol, Newborn, Intestine, Murine experimental model

## Abstract

**Background:**

Necrotizing enterocolitis (NEC) is a severe inflammatory bowel disease in neonates. Our group has developed an experimental model of NEC, with an effectiveness of 73%. Cannabidiol (CBD) is an innovative treatment for neonatal cerebral hypoxic-ischemic pathologies due to its neuroprotective effect, as a potent anti-inflammatory and reducing oxidative stress substance.

Our aim was to evaluate the effect of CBD on intestinal lesions in an experimental model of NEC.

**Results:**

Mortality and intestinal histological damage was significantly lower in the CBD group compared to the rest (*p*<0.05), establishing CBD as a protective factor against the development of NEC (OR=0.0255; 95% CI=0.0015-0.4460). At IHQ level (TUNEL technique), a lower cell death rate was also observed in the CBD group compared to the VEH group (*p*<0.05).

**Conclusions:**

In our experimental model, intraperitoneal CBD acts as a protective factor against NEC, resulting in less histological damage and a lower rate of intestinal cell death.

## Background

NEC is a severe inflammatory bowel disease in neonates affecting 5-12% of very low birth weight infants, requiring surgery in 20-40% of cases and being fatal in 25-50% of patients [1]. In neonatal intensive care units it is mainly a pathology of prematurity, affecting 10-12% of neonates under 1500 grams, with a mortality of 10-30% [2, 3].

In our country, according to the SEN1500 registry in 2018, NEC occurred in 5.1% of children under 1500 g, requiring surgical treatment in 61.8% with a mortality of 10-30% [4].

NEC is not only a clinical but also an economic problem. Newborns requiring surgery are hospitalised an average of 60 days longer than those without NEC [5]. NEC therefore accounts for a large part of the economic burden associated with preterm birth; in fact, the average total cost of treatment per patient is estimated at $500,000, with the total cost per year in the United States estimated to be between $500 million and $1 billion [5–8].

Despite decades of research, the pathogenesis of the disease remains unclear, and prevention strategies are limited and ineffective [9].

NEC is characterised by ischaemic necrosis and inflammation of the gastrointestinal tract with destruction of the mucosal lining in the early stages. In advanced stages, transmural necrosis of the intestinal wall and often perforation occurs [10].

Although the aetiology of NEC is multifactorial, some factors are of particular importance, such as prematurity, asphyxia, formula feeding and bacterial infection, as well as episodes of ischaemia/reperfusion [11].

The ischaemia/reperfusion (I/R) episodes are one of the most important aetiological factors for NEC [11]. I/R increases oxidative stress and inflammation, leading to necrosis and apoptosis of intestinal cells [12]. Apoptosis has been demonstrated in the apical zone of intestinal villi of children with NEC and in histological samples harvested at the time of resection of the affected intestine [13]. The oxidative stress agents that scavenge free radicals (RLO) such as superoxide dismutase (SOD) [14] or inhibit their formation by xanthine oxidase (XO), such as allopurinol, prevent intestinal injury caused by I/R [15]. In the inflammation tumour necrosis factor α (TNF-α) [16], interleukin-18 (IL-18) [17] and platelet activating factor (PAF) [16] play an important role in the development of NEC [11]. Hypoxia and/or ischaemia, which are important risk factors for the development of NEC [11], induce an inflammatory cascade involving several cytokines [18].

Treatment nowadays generally includes broad-spectrum antibiotics, absolute diet and fluid support [6, 7, 19]. Surgical intervention is necessary if the condition progresses. Unfortunately, the lack of specific and effective treatment strategies for NEC is a major contributor to the high overall morbidity and mortality in patients with this disease [20].

Cannabidiol (CBD) is the main non-psychostimulant component of *Cannabis sativa* [21, 22]. In the case in question, in terms of diseases of the digestive system, on studying the possible effects of cannabinoids on the gastrointestinal tract, it has been shown to include all the components of the endocannabinoid system, which implies a possible strong impact of substances such as cannabidiol [21]. Its multiple mechanisms of action include a potent anti-inflammatory and antioxidant effect [22]. Our group succeeded in developing a model of necrotising enterocolitis in the newborn Wistar rat with an effectiveness of 73% in the presentation of intestinal lesions. The murine model described by our group associates feeding of newborn rats with formula milk together with lipopolysaccharide (LPS), and episodes of hypoxia with hypothermia [23].

## Methods

The protocol was carried out according to the ethical basis for research in animal experimentation. The Institutional Animal Care and Use Committee of the Hospital Clínico San Carlos approved the project (#280790000088; PROEX 196.4/21; CEEA 21/0 04-III).

### Experimental design

We used newborn Wistar rats (*N*=80) after controlled gestation and delivery, separating the newborn pups from the mother in the three study groups, avoiding breastfeeding. For three days and every 6 hours (12 feedings in total), they were given a hypertonic milk formula prepared with 10 ml of humanised 60/40 milk (Blemil plus 1®) in 30 ml of Esbilac® dog supplement, through a 1 Fr catheter [23]. After each milk feeding, we induced a hypoxic episode that lasted 10 minutes (95% N2 and 5% O2) associated with hypothermia was induced.

We weighed the infants before each feeding and randomly divided them into 3 groups.

Sham group with the newborns getting breastfed with the mother (*N*=14), of which 12 completed the 72-hour study period (mortality = 14%). Vehicle group (VEH) (*N*=38), to which the NEC model is induced (formula milk, LPS, hypoxia and hypothermia) and injected with saline. Only 3 completed the 72-hour study period (mortality = 92%). NEC was induced by two feedings of milk with LPS (*Escherichia coli* 0127:B8 lipopolysaccharide) (feeding 2 and feeding 6) by mixing the formula milk with an amount in ml of LPS (mg/ml) at a dose of 4 mg/kg. Finally, the treatment group (CBD) (*N*=28), to which the NEC model is induced (formula milk, LPS, hypoxia and hypothermia) and are treated with an intraperitoneal injection of cannabidiol (CBD) (LGC, DR-EHRENSTORFER, G1257704) prior to feeding number 3. Nine completed the 72 hour study period (mortality = 68%). The CBD injection (0.05 ml) consisted of a 10 mg/kg dose dissolved in a 1:1:18 ratio (CBD stock dissolved in ethanol; Kolliphor®EL; Mein Sodium Chloride 0.9%).

The amount of milk provided correlates with their basal needs (4 kcal/g) [24] and in relation to the weight of the calves, so that: Pups weighing less than 5 grams are given 0.3 ml in the first 4 feedings and then each 4 feedings increase 0.05 ml. Pups weighing more than 5 grams are given 0.35 ml in the first 4 feedings and then each 4 feedings increase 0.05 ml.

### Clinical situation

To assess the welfare of the animals during the process, their appearance, natural activity, response and colour (Table 1) are analysed and studied before and after each feeding. This way, a value of 2 requires more exhaustive monitoring and a value of 3 requires immediate euthanasia of the newborn to avoid prolonging its suffering.

**Table 1:** Clinical sickness score for the assessment of neonatal rat clinical status [24]

**Appearance**
0 = Tonic and well-hydrated
1 = Slimmer, but still tonic and hydrated
2 = Skinny, floppy and dehydrated
3 = Gasping and in agony
**Natural activity**
0 = Moving normally
1 = Able to wriggle if put supine
2 = Not able to wriggle if put supine
3 = Not moving limbs and lying still
**Response to touch**
0 = Alert
1 = Responding to mid stimulation
2 = Responding to vigorous stimulation
3 = Unresponsive notwithstanding vigorous stimulation
**Body colour**
0 = Pink
1 = Pale (just the extremities)
2 = Pale (whole body)
3 = Grey

### Macroscopic appearance and histological study of small and large intestine

The neonates of the 3 groups were euthanised 6 hours after the last feeding. A longitudinal incision was made along the abdomen and the gastrointestinal tract was removed from the rectum to the esophagus. We then assessed the condition of the intestine, and the degree of involvement or degree of enterocolitis were assessed according to its consistency, colour and dilatation (Table 2).

**Table 2:** Scoring system for macroscopic gut assessment [24]

**Gut consistency**
0 = normal
1 = moderately friable
2 = extremely friable (jelly like)
**Gut colour**
0 = normal
1 = patchy discoloration
2 = extensive discoloration
**Gut dilatation**
0 = no dilatation
1 = patchy dilatation
2 = extensive dilatation

### Histology

The samples were fixated in formaldehyde (FA) for 3 days and the tissue was processed by dehydration. We then embedded them in paraffin. We made histological sections with a microtome (Leica Biosystems, Nussloch, Germany) at 4 microns and stained with haematoxylin - eosin.

**Fig. 1:**
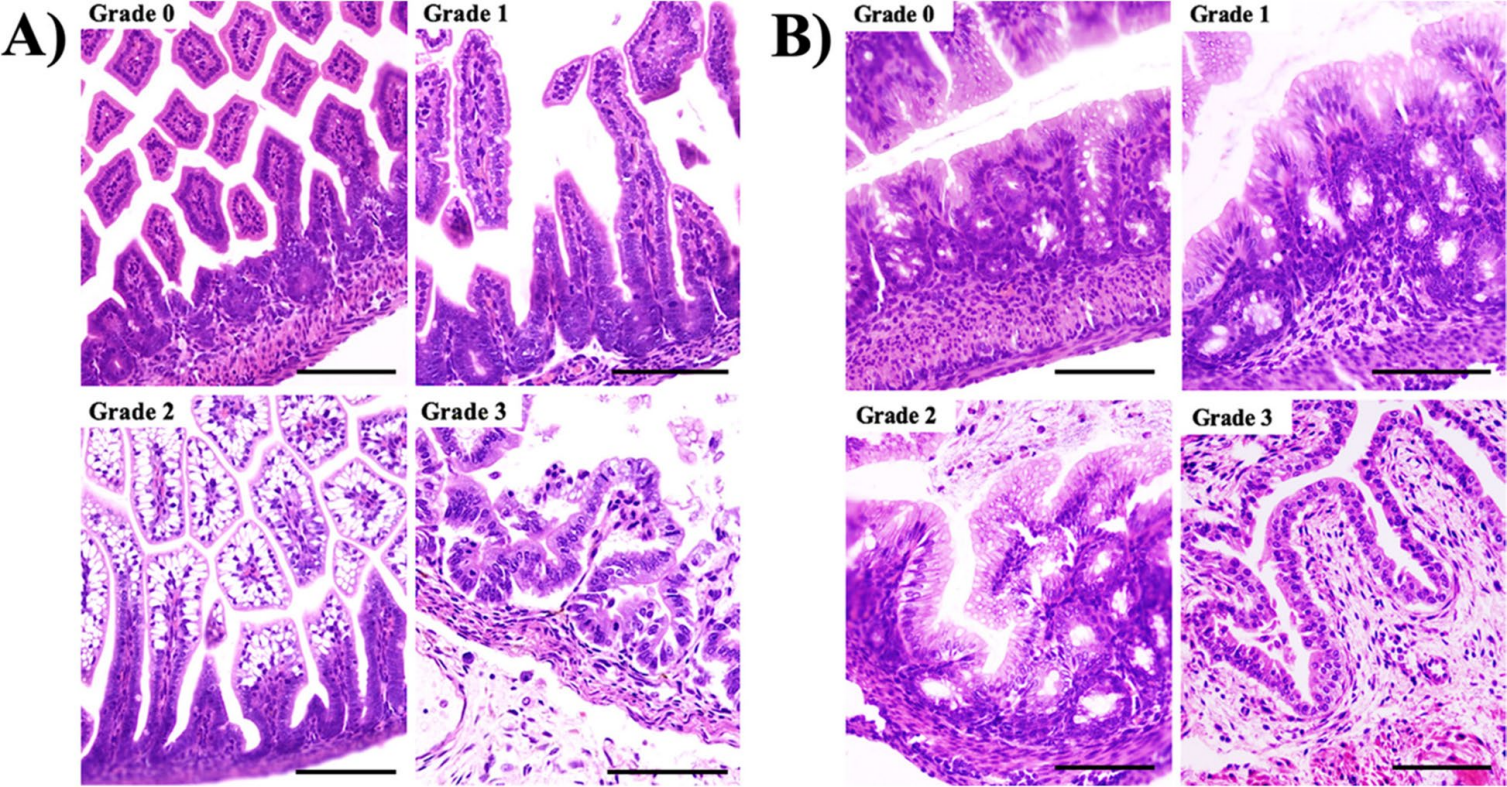
Pictures of histological sections (x40) of the small intestine (**A**) and large intestine (**B**) with haematoxylin-eosin staining of the 4 degrees of histological damage due to enterocolitis. *Grade 0 = healthy intestine; Grade 1 = detachment of enterocytes and mild wall separation; Grade 2 = detachment of enterocytes and severe wall separation; Grade 3 = epithelial villous detachment/cell rupture.* Scale 100 microns

We used the assessment system modified by Nadler [24, 25] to determine the degree of necrosis of the intestine or the degree of NEC damage, with 0 being the least affected or healthiest state of the intestine and 3 the most affected (Fig. 1): 0 = healthy intestine; 1 = villous enterocyte detachment and mild wall separation; 2 = villous enterocyte detachment and severe wall separation; 3 = villous epithelial detachment/cellular rupture. In this case, each intestinal specimen is analysed according to the portions of the small intestine (Fig. 1A) and large intestine (Fig. 1B) in the histological section. This way, we can have, for example, a total of 30 portions of small intestine and 5 portions of large intestine in one slice. According to this total, we evaluate how many portions of each intestine present each of the degrees of NEC damage, obtaining a percentage. We work with the latter to obtain an average of the degrees of NEC between study groups. This way, a greater or lesser effect of CBD can be evaluated in the NEC model, i.e. grades 0 and/or 1 of NEC damage indicate less damage to the intestine, while grades 2 and/or 3 of NEC damage indicate greater damage to the intestine.

### Immunohistochemistry

We used a confocal microscope AFV1000, OLYMPUS, to obtain the photos. The analysis of the photos was carried out with Fiji software.

#### TUNEL (cell death)

The 4 micron sections follow a deparaffinisation process (Xylol, Ethanol 100%; 96%; 80%; 70% and Sodium Chloride (NaCl) 0.9%). The DeadEnd™ Fluorometric TUNEL System kit, Promega, G350, is then used. This technique identifies the apoptotic nuclei of the tissue cells, observed at a wavelength of 488 nm (green), with respect to the total number of nuclei present. The latter are distinguished by the nuclear marker DAPI at a dilution of 1:1000, observed at a wavelength of 465 nm (blue).

In this case, the absolute number of apoptotic nuclei in the tissue is compared between groups.

#### Iba-1 (macrophages)

The 4 micron sections follow a dewaxing process (Xylol, Ethanol 100%; 96%; 70% and distilled water), an epitope unmasking with Sodium Citrate (Na3C6H5O7), ph=6, and subsequent washes. For the unmasking, sodium citrate was preheated in a glass jar in a microwave oven (700W) at half power for 4 minutes, in an amount sufficient to cover the slides. The slides were then inserted and heated for 6 minutes.

We used the primary anti-Iba-1 rabbit antibody (for IHC, Wako, 019-19741) at 1:300 dilution and the secondary antibody Alexa-Fluor 594 goat anti-rabbit IgG (INVITROGEN, A11012) at 1:200 dilution, observed at a wavelength of 594 nm, to mark the intestinal macrophages. Additionally, we used the nuclear marker DAPI at 1:1000 dilution observed at a wavelength of 465 nm to distinguish individual cells.

The NEC lesion is identified by quantifying the number of cells and the integral of the fluorescence intensity of active macrophages (Iba-1) with respect to the number of cells and the integral of the fluorescence intensity of DAPI, taking the latter as a percentage of 100%. In this case, our hypothesis assumes that the greater the lesion, the greater the inflammatory response and the lower the number and intensity of macrophages, since in this case the presence of polynuclear neutrophils would increase, displacing the macrophages of the lamina propria.

### Statistical analysis

The data were analysed using Graphpad software: The analysis of variance ANOVA was used for weight, mortality and immunohistochemistry results to compare data from all groups. The Chi-square test was used for the degree of macroscopic damage (consistency, colour and dilatation). The Kruskar Wallis and Mann-Whitney U tests were used for histological lesion severity. The odds ratio (OR) of CBD was studied to assess its possible protective effect against NEC.

## Results

### Clinical situation

For 72 h, the condition of the hatchlings was recorded, assessing their appearance, natural activity, perception and colour (Table 1). At 48 hours, the effect of NEC was observed: the hatchlings became pale with signs of dehydration and their abdominal organs became visible due to swelling.

### Weight and mortality

**Fig. 2:**
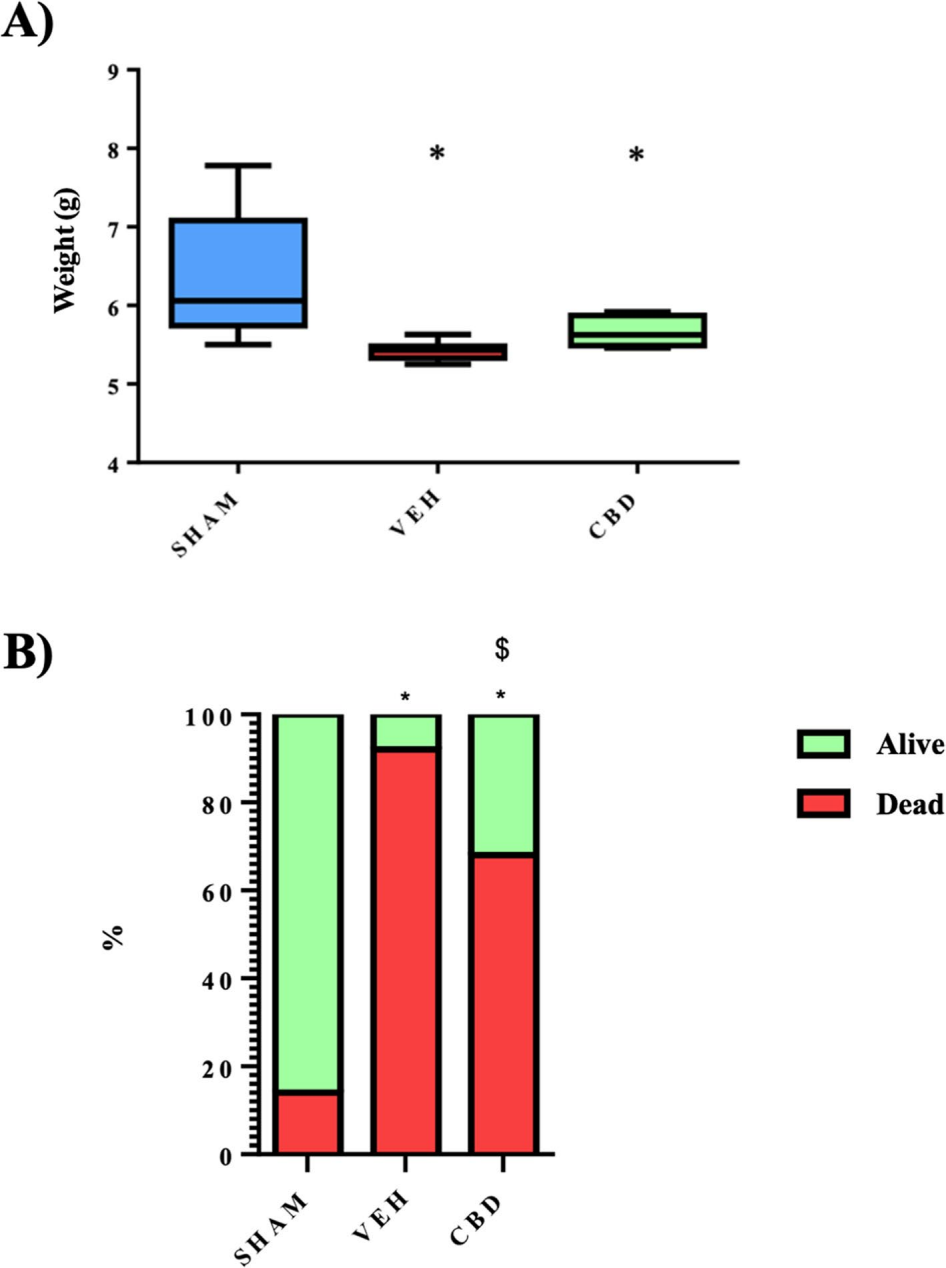
**A** Comparison of weight variation in each of the groups throughout the model (*p* < 0.05). **B** Comparison of the variation in mortality between hatchling groups throughout the model (*p* < 0.05). The green colour represents those sacrificed at 72 hours while the red colour represents those that die throughout the model. * significant difference compared to SHAM, $ significant difference compared to VEH

Throughout the 12 feedings over the 72 hours of the model, the neonates had varying weights (Fig. 2A). On average, the SHAM group weighed 6.35 g, while the CBD group weighed 5.66 g and the VEH group 5.42 g. Significant differences were observed between the SHAM group and the CBD and VEH groups but no significant differences were observed between the CBD and VEH groups (Fig. 2A).

Mortality in the model, or VEH group, was 92%, including deaths before the last catheter was injected and deaths due to catheter misinsertion at the injection, leading to oesophageal perforation (Fig. 2B). Mortality in the CBD group was lower at 68% (Fig. 2B). Mortality analysis showed significant differences between SHAM with CBD and VEH and between the CBD and VEH group (Fig. 2B).

### Macroscopic appearance and histological study of small and large intestine

In the macroscopic analysis of the intestine, no significant differences were observed between the SHAM group and the CBD and VEH groups for consistency and dilatation. However, there were significant differences between the SHAM and VEH groups.

**Fig. 3:**
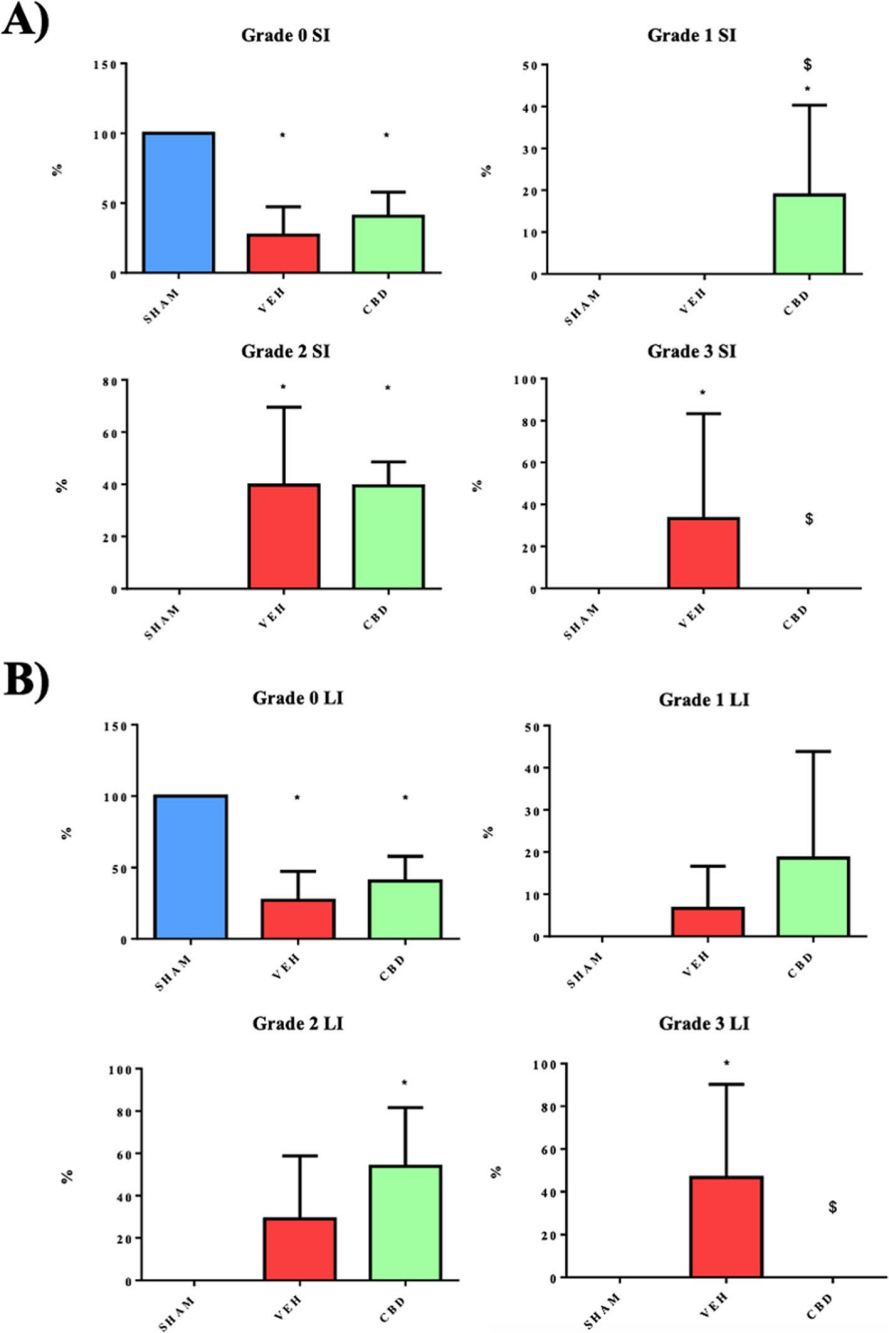
**A** Comparison of the variation in the values of consistency, colour and dilatation of the intestine at extraction for each of the study groups (*p* < 0.05)*.* **B** Comparison of the variation of NEC grades, observed histologically with Haematoxylin-Eosin staining, of the small intestine in each of the study groups **C**) Comparison of the variation of NEC grades, observed histologically with Haematoxylin-Eosin staining, of the large intestine in each of the study groups. *P* value < 0.05. * significant difference compared to SHAM, $ significant difference compared to VEH

The statistics for the grades of histological damage or injury by NEC vary in both intestines, as shown in Fig. 3A and B. The significant differences that stand out the most are mainly observed in grade 0 of the SHAM group with respect to VEH and CBD and, subsequently, the differences between the latter two in grade 1 (small intestine) and for grade 3 (in both intestines).

### Immunofluorescence

#### TUNEL (Fig. 4A)

**Fig. 4:**
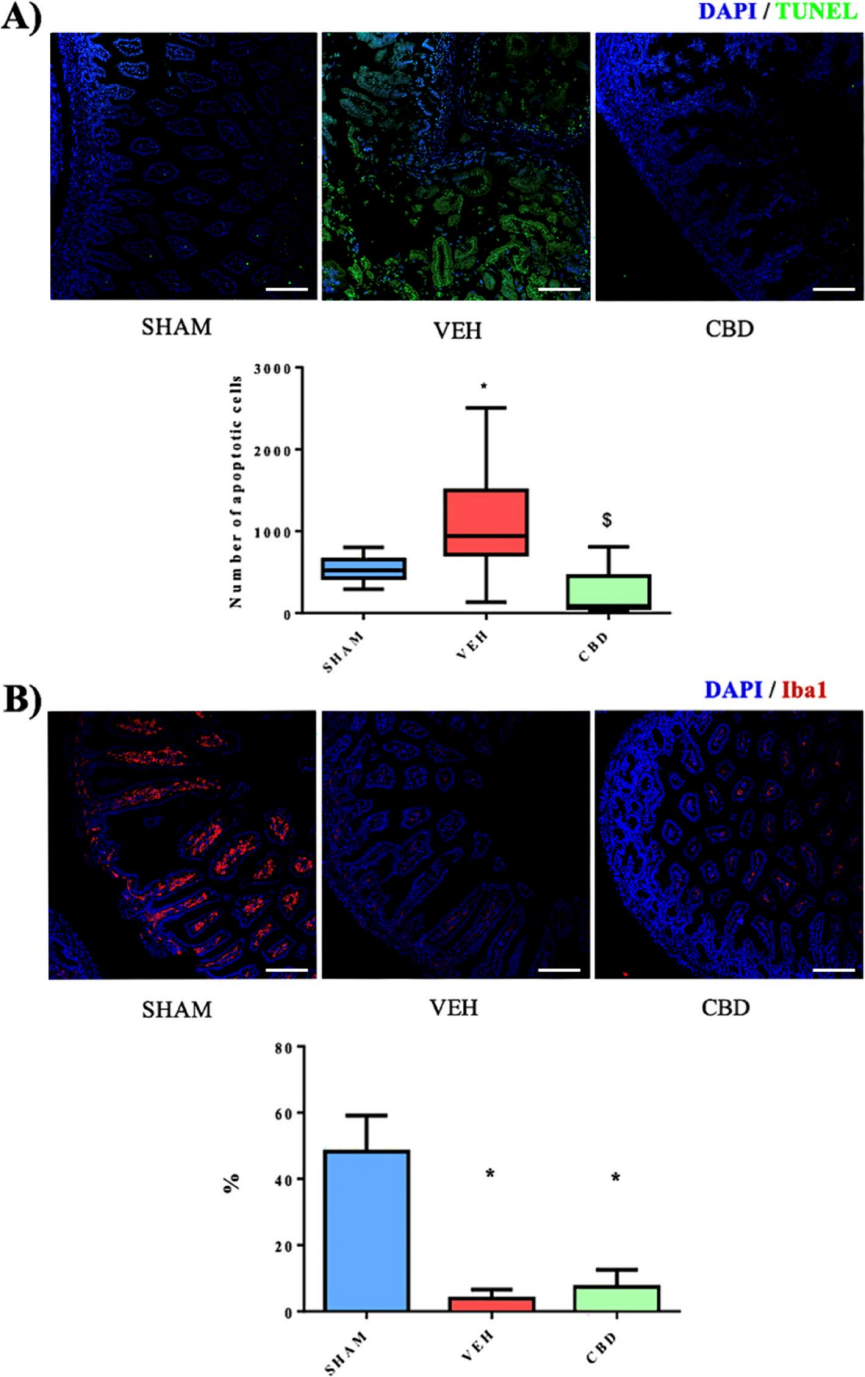
A Immunofluorescence (x20) with DAPI (cell nuclei in blue) and TUNEL (apoptotic cell nuclei in green) antibodies of SHAM, VEH and CBD groups and comparison of the variation of the absolute number of apoptotic cells of the 3 study groups at 72h (*p* < 0.05). Scale 100 microns. B Immunofluorescence (x20) with DAPI antibodies (cell nuclei in blue) and Iba-1 (macrophages in red) of the SHAM, VEH and CBD groups and comparison of the variation of the percentage of Iba-1 with respect to DAPI of the 3 study groups at 72h (*p* < 0.05). Scale 100 microns. * significant difference compared to SHAM, $ significant difference compared to VEH

According to the results, there are significant differences in the percentage of fluorescence of apoptotic nuclei in the tissue between the SHAM and VEH groups but not with the CBD group. There are also significant differences between the VEH and CBD groups.

#### Iba-1 (Fig. 4B)

According to the results there are significant differences in the percentage of Iba-1 fluorescence with respect to DAPI between the SHAM group and the VEH and CBD groups, but not between the VEH and CBD groups.

#### Odds ratio (OR)

Treatment with CBD as a protector in this NEC model has an OR of 0.0255 with a 95% confidence interval (95% Confidence interval (CI)) between 0.0015-0.4460 values. The *P* value corresponds to 0.0120.

## Discussion

NEC is a devastating intestinal disease that affects 10-12% of preterm infants with a mortality between 10% and 30% [2, 3], and whose pathogenesis is still unclear. Our team has been able to reproduce this disease with an effectiveness of 73% [23] and, on this occasion, with an effectiveness of 100% and a mortality of up to 92% in Wistar rat neonates born with LPS, hypoxia and hypothermia.

This murine model has been developed based in other work such as the study by Miyake et al. [26] in 5-day-old C57BL/6 mice, where NEC is induced by feeding milk formula 3 times a day and only one LPS intake per day (dose 4mg/kg), via catheter/probe combined with 10 min hypoxia episodes 3 times a day (at 95% N_2_ and 5% O_2_)

Of note is the study by Zani et al. [24] using premature offspring of rats (Sprague-Dawley) obtained via caesarean section. Subsequently, NEC is induced by feeding milk formula 3 times daily, including LPS (dose 4 mg/kg) at the first feeding on day 1 and 2 of the model, via catheter/probe together with 10 min hypoxia episodes before each feeding (at 95% N_2_ and 5% O_2_)

Our model is very similar to the study by Zani et al. [24] with the difference that our offspring are Wistar neonates, since offspring obtained by caesarean section are excessively premature and pose many management difficulties in our environment. Even so, our model has shown adequate effectiveness [23] and would follow a procedure similar to that of other studies [27–30].

Variations have been described modifying the factors that are considered to be etiopathogenic in NEC [29, 30]: Hypoxia in % N_2_ and O_2_, the number of times and the time it is induced (from 50s to 10 min). Other models even Re oxygenate after hypoxia [31]; Hypothermia (if done), at 4°C in combination with hypoxia or after hypoxia with a time of 3 to 10 min [24–30, 32–37]; Administration (if any) of LPS (at doses of 1-20 mg/kg) [24, 26, 27, 32–34, 36].

Once the model and its effectiveness had been defined, the non-psychostimulant cannabinoid cannabidiol or CBD was chosen as a therapeutic option, due to its experimental use in several murine models of LPS-induced intestinal inflammation, in which it has shown multiple positive effects: reduction of histochemical damage and the promotion of positive regulation of the inflammatory response pathway (cytokines, chemokines and oxidative stress markers) [21]. Specifically, the preventive effect of CBD was observed in a Sprague-Dawley rat model of LPS-induced intestinal inflammation [21]. In this case, the protective effect of CBD was observed, reducing histopathological damage, TNF-α and Interleukin 6 (IL-6) levels along with the restoration of smooth muscle and its myoelectric activity [21].

Other studies on inflammatory bowel diseases have shown that endocannabinoids, thanks to the intestinal endocannabinoid system, are capable of inhibiting extensive pathways that release pro-inflammatory mediators such as Interleukin beta 1 (IL-ß1), multiple cytokines, the well-known TNF-α and nitric oxide, which are ultimately the main causes of these disorders [31, 38, 39]. Again, CBD specifically presents itself as a therapeutic approach to these conditions, as it not only has anti-inflammatory, antioxidant and anti-apoptotic properties for the central nervous system, but is also a major anti-inflammatory in the gut, with one of its main effects being the inhibition of neutrophilic chemotaxis [27, 38, 40, 41]. A case in point is seen in the Swiss mouse study by De Filippis et al. [27], testing the effect of CBD (at a dose of 10mg/kg) against acute infection and inflammation as a result of enteroglial protein S100B in combination with LPS (at a dose of 20mg/kg). CBD triggers inhibition against mainly neutrophil recruitment and also reduces the presence of macrophages, as represented by the intestinal macrophage-specific marker MAC3 signal. Consequently, there was a reduction in TNF-α kinase expression and intestinal histological damage observed with low caspase-3 expression.

Other studies with experimental models of NEC have assessed the protective effect of different substances such as Lactobacillus [35] vitamin B5 [42], colchicine [43] Teduglutin (glucagon-like peptide-2, GLP-2) [34] allopurinol [17] or daikenchuto (a mixture of medicinal herbs) [44]. However, none of these treatments have been successfully translated to the clinic, so there is still a need to investigate new possibilities. In this respect, cannabidiol is a very promising candidate.

Thus, the effect of CBD was studied in our NEC model, demonstrating its protective effect with an odds ratio (OR<1) value of 0.0255.

The dose of CBD used in this model is 10 mg/kg and is injected only once after induction of NEC. The dose was selected based on our group's previous studies of neonatal hypoxic-ischaemic brain damage [1–5, 25, 36, 37, 45].

The results show that, in the case of weight, the SHAM group increases moderately to almost 8 grams on the last feeding of the third day, i.e. corresponding to the weight of the usual control group [35, 37]. However, the VEH and CBD groups decrease or maintain the newborn weight, between 4 and 5 grams, with no differences between them. The same occurs in the study by Weis et al. [36], whose treatment consists of the implantation of human placental stem cells (hPSC), observing that the weight of the group with NEC and the group with NEC and treated decreases as the model progresses, worsening due to the disease itself, with no significant statistical difference to support this (*p*<0.03). On the other hand, in other studies using Infliximab [35] (TNF-α antagonist) and Tocilizumab [37] (Interleukin-6 signal inhibitor) as treatment, there are significant differences between the weights of the NEC group and the NEC and treatment group, with a *p*-value of *p*<0.001 and *p*<0.0001 respectively.

Although the calculations of volume and calories administered in the intakes of the administered milk meet the requirements established for the weight of newborns (4 Kcal/g per day) [46], the severity of the NEC manifests a clinical impact that has not been reversed by CBD treatment, which may be a sign of an underestimation of caloric requirements.

In the macroscopic study of the tissue and compared to the healthy intestine of the SHAM group, the only notable difference between the VEH and CBD groups was the difference in colour when the viscera were removed, as the consistency and dilatation corresponded to severe NEC.

The mortality of the model (92%) includes the death caused by oesophageal perforation estimated in this model (5%). In this aspect, the effect of CBD is clear, as it manages to reduce mortality by almost 24% (68%).

The analysis of the histological NEC damage grade of the groups also shows changes with respect to the model. Compared to the SHAM group, with a grade 0 for both the small and large intestine, due to its normal and healthy state, the VEH group presents, in most of the tissue, a grade 2 and 3 NEC injury. On the other hand, the CBD group has mostly grade 1 and 2 lesions.

The main difference in our method of assessment compared to other studies [33–37] is that we aim to observe the progressive change of the tissue and thus the progressive effect of the treatment (CBD). This is why we do not label as total NEC those tissues with grade 2 or higher histological involvement by NEC but quantify the percentage of grades present in the tissue based on the total present. In other studies, such as that of Feng et al. [33], where a heparin-binding epidermal growth factor (HB-EGF) is used as treatment, that of Nakame et al. [34], whose treatment consists of a trophic gut hormone, glucagon-like peptide 2 (GLP-2), and the studies by Tayman et al. [35], Weis et al. [36] and Yarci et al. [37] that we are already familiar with, the tissue with grade 2 or higher NEC damage is evaluated as NEC. All these studies also show statistically significant differences between the NEC and NEC with treatment groups. Specifically in the studies by Feng et al. [33] and Nakame et al. [34], the reduction in damage is approximately 25%, with a *p*-value of *p*<0.05. In the study by Weis et al. [36] this reduction is less, 9% with a *p*-value of *p*<0.0001, while in the studies by Tayman et al. [35] and Yarci et al. [37] this reduction is not quantified in percentage, but these data show a significant difference with *p*-values of *p*<0.002 and *p*<0.001 respectively.

For the immunohistochemical study, the technique for identifying or marking cell apoptosis (TUNEL) and the technique for identifying microglia and macrophages (Iba-1) have been used. On the one hand, the TUNEL technique is highly effective in marking cell death in various tissues [46, 47]. On the other hand, the Iba-1 technique, which, in addition to being used in studies of the nervous system (microglia), has been frequently used for the general identification of macrophages in intestinal tissue [48].

In the case of our results, we observed a high level of cell death or apoptosis in the intestinal tissue of the VEH group, in contrast to the SHAM and CBD groups. Specifically, there is a wide range of variation in the tissue of the CBD group, being similar to the SHAM group, which again shows the protective effect, in this case immunohistochemical, of CBD. Similar studies observe a reduction in histological damage due to a reduction in the number of apoptotic cells per area [33, 35, 37].

In other studies, such as those by Feng et al. [33], Tayman et al. [35] and Yarci et al. [37], of which we are aware, significant differences are also observed with the TUNEL technique between the groups with NEC and those with NEC and treatment. In other words, a reduction in histological damage is observed due to a reduction in the number of apoptotic cells per area. In this case the first study uses a *p*-value of *p*<0.05, like us, while the studies of Tayman et al [35] and Yarci et al [37] use *p*<0.001 and *p*<0.002 respectively.

Regarding the Iba-1 study, we observed that the SHAM group shows high fluorescence and a large amount of macrophages, on average 47.84%, with the lamina propria being the most fluorescent layer, i.e., the data correlates with the normal, healthy state of the intestine. However, the VEH and CBD groups show very low Iba-1 fluorescence, with an average of 2.85% and 6.61% respectively.

Our initial hypothesis assumed a progressive decrease in the Iba-1 signal (macrophages) as intestinal damage and inflammation sign. As such the group with the highest signal, after the results, corresponds to SHAM, which confirms our hypothesis. Thus, the VEH and CBD groups would be the ones with the least Iba-1 signal due to the damage and inflammation produced by the NEC. This is due to the displacement of macrophages from the lamina propria by polynuclear neutrophils formed as a result of the immune system's response to necrotic damage [27].

Although there are no significant differences for the Iba-1 signal between the VEH and CBD groups, in the case of VEH this may be due to the extreme necrosis of the tissue demonstrated by the grade 3 NEC damage (Fig. 3) and the apoptotic death of the tissue demonstrated by the TUNEL technique (Fig. 4A). On the other hand, the low Iba-1 signal in the CBD group may be due to the effect of CBD itself on the immune system, as the histological damage grades show that it has low levels of NEC (Fig. 3) correlated with low apoptotic cell death (Fig. 4A). In other words, the CBD group is in better histopathological condition and the low Iba-1 fluorescence may be due to the anti-inflammatory effect of CBD itself [49]. These results are in line with those shown in the study by De Filippis et al. [27].

## Conclusions

In this paper we present the effect of a low dose of CBD on the NEC model developed by our team. The data show the protective effect of CBD on factors such as mortality, degree of histological damage and tissue cell death (TUNEL). However, in other factors or variables such as weight along the model, macroscopic analysis and immune response (Iba-1 immunofluorescence) the data do not indicate sufficient difference between the VEH and CBD groups.

Although the initial hypothesis on the protective effect of CBD has been confirmed (OR<1), our results seem to indicate that future studies are needed to evaluate the dose and schedule of CBD administration in our neonatal rat model of NEC. These data open a clear hope for the treatment of clinical NEC.

## Data Availability

In the manuscript.

## Abbreviations

CBD: Cannabidiol
CI: Confidence interval
FA: Formaldehyde
GLP-2: Glucagon-like peptide-2
HB-EGF: Heparin-binding epidermal growth factor
hPSC: Human placental stem cells
IHQ: Immunohistochemical
IL-6: Interleukin 6
IL-18: Interleukin-18
I/R: Ischaemia/reperfusion
LPS: Lipopolysaccharide
NaCl: Sodium Chloride
NEC: Necrotizing enterocolitis
OR: Odds ratio
PAF: Platelet activating factor
RLO: Free radicals
SOD: Superoxide dismutase
TNF-α: Tumour necrosis factor α
XO: Xanthine oxidase
